# Kaczmarz Iterative Projection and Nonuniform Sampling with Complexity Estimates

**DOI:** 10.1155/2014/908984

**Published:** 2014-12-14

**Authors:** Tim Wallace, Ali Sekmen

**Affiliations:** Department of Computer Science, Tennessee State University, 3500 John A. Merritt Boulevard, Nashville, TN 37209-1500, USA

## Abstract

Kaczmarz's alternating projection method has been widely used for solving mostly over-determined linear system of equations *A* **x** = **b** in various fields of engineering, medical imaging, and computational science. Because of its simple iterative nature with light computation, this method was successfully applied in computerized tomography. Since tomography generates a matrix *A* with highly coherent rows, randomized Kaczmarz algorithm is expected to provide faster convergence as it picks a row for each iteration at random, based on a certain probability distribution. Since Kaczmarz's method is a subspace projection method, the convergence rate for simple Kaczmarz algorithm was developed in terms of subspace angles. This paper provides analyses of simple and randomized Kaczmarz algorithms and explains the link between them. New versions of randomization are proposed that may speed up convergence in the presence of nonuniform sampling, which is common in tomography applications. It is anticipated that proper understanding of sampling and coherence with respect to convergence and noise can improve future systems to reduce the cumulative radiation exposures to the patient. Quantitative simulations of convergence rates and relative algorithm benchmarks have been produced to illustrate the effects of measurement coherency and algorithm performance, respectively, under various conditions in a real-time kernel.

## 1. Introduction

Kaczmarz (in [1]) introduced an iterative algorithm for solving a consistent linear system of equations *A* **x** = **b** with *A* ∈ *ℝ* ^*M*×*N*^. This method projects the estimate **x** ^*j*^ onto a subspace normal to the row *a*_*i*_ at step *j* + 1 cyclically with *i* = *j*(mod⁡*M*) + 1. The block Kaczmarz algorithm first groups the rows into matrices *A*_1_, *A*_2_,…, *A*_*k*_ and then it projects the estimate **x** ^*j*^ onto the subspace normal to the subspace spanned by the rows of *A*_*i*_ at step *j* + 1 cyclically with *i* = *j*(mod⁡ *k*) + 1. Obviously, the block Kaczmarz is equivalent to the simple Kaczmarz for *k* = *M*. The Kaczmarz method is a method of alternating projection (MAP) and it has been widely used in medical imaging as an algebraic reconstruction technique (ART) [2, 3] due to its simplicity and light computation. Strohmer and Vershynin [4] proved that if a row for each iteration is picked in a random fashion with probability proportional with *ℓ*_2_ norm of that row, then the algorithm converges in expectation exponentially with a rate that depends on a scaled condition number of *A* (not on the number of equations). Needell (in [5]) extended the work of [4] for noisy linear systems and developed a bound for convergence to the least square solution for *A* **x** = **b**. Needell also developed a randomized Kaczmarz method that addresses coherence effects [6], and she analyzed the convergence of randomized block Kaczmarz method [7]. Chen and Powell (in [8]) consider a random measurement matrix *A* instead of random selection of measurements. Galántai (in [9, 10]) provides convergence analysis for block Kaczmarz method by expanding the convergence analysis (based on subspace angles) of Deutsch [11]. Brezinski and Redivo-Zaglia (in [12]) utilizes the work of Galantai for accelerating convergence of regular Kaczmarz method.

The work of this paper endeavors to make the following contributions.

- Research on regular and randomized Kaczmarz methods appears disconnected in the literature. Even though convergence rates have been studied separately, the link between them has not been explored sufficiently.
- A new randomization technique based on subspace angles has been developed which indicates an advantage with coherent data measurements.
- A further method is introduced which orthogonalizes the subspace blocks in order to mitigate the coherency. Convergence is consistent with statistical expectations from theory and simulations.
- The effects of measurement coherence are observed in the literature and illustrated in our simulations with norm and angle based iteration randomization.
- A broader review and mathematical analysis of common methods is presented from both statistical and deterministic perspectives.
- Numerical simulations are provided to illustrate the typical effects of nonuniform sampling coherency upon convergence of Kaczmarz methods.
- Kaczmarz inversions versus matrix size were performed to allow comparison of the relative convergence rates of various well-known methods using typical hardware and software. The results show relative computational complexities of common methods for simple and randomized Kaczmarz, including the randomized Kaczmarz orthogonal block.

## 2. Methods and Materials

Data inversion and reconstruction in computed tomography is most often based upon the iterative Kaczmarz algorithm due to the *O*(*N* ^2^) performance. First, in this section, given the number of methods currently in the literature, a broad but extensive overview of the mathematical theory for the more common methods is provided, such as simple, block, and randomized Kaczmarz. Where possible, the convergence results are compared from both random and deterministic perspectives to demonstrate similar results and convergence analysis methods. The concept of subspace projections is reviewed and the connection to iteration is noted.

Next, two new methods are proposed and analyzed in the context of coherent data measurements. These methods allow the algorithm to adapt to the changing environment of the sampling measurement system, in order to mitigate coherency.

Simulated methods for data acquisition under uniform and nonuniform X-ray beam measurements are included, and convergence results are computed comparing simple and random row selection methods.

Lastly, after the algorithm methods section, a brief overview of the methods used to obtain the complexity estimates is presented. Methods include using common software and hardware under dedicated kernel conditions. Simulations for Kaczmarz convergence and complexity were performed using Octave software.

### 2.1. Convergence of Regular Block Kaczmarz Method

Let **x** ^*^ be the solution of consistent *A* **x** = **b**, where *A* ∈ *ℝ* ^*M*×*M*^ is full column rank. Let *A* be row-partitioned as {*A*_1_,…, *A*_*k*_} where *A*_*i*_ ∈ *ℝ* ^*M*_*i*_×*M*^. Then, the simple block Kaczmarz update is as follows:

(1) $$\begin{array}{c} x_{j+1}=x_{j}+A_{i}^{T}{\left(A_{i}A_{i}^{T}\right)}^{-1}\left(b_{i}-A_{i}x_{j}\right)\;i=j\left(\mathrm{mod}k\right)+1, \end{array}$$

where **b**_*i*_ is the section of **b** that corresponds to the rows of *A*_*i*_. Note that since *A*_*i*_ is full row rank, *A*_*i*_^*T*^(*A*_*i*_ *A*_*i*_^*T*^)^−1^ is the right pseudo-inverse of *A*_*i*_. This is equivalent to

(2) xj+1=xj+AiTAiAiT−1Aix∗−Aixj,xj+1−x∗=xj−x∗−AiTAiAiT−1Ai(xj−x∗).

Note that *A*_*i*_^*T*^(*A*_*i*_ *A*_*i*_^*T*^)^−1^ *A*_*i*_ is the projection matrix for projection of the range of *A*_*i*_^*T*^:

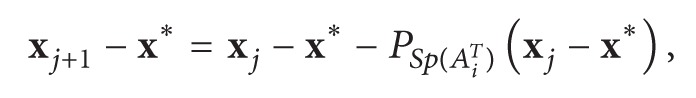
(3)

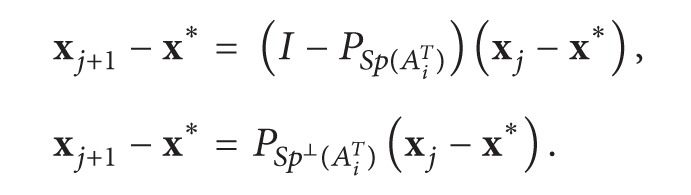
(4)
For one cycle of the blocks,

(5) $$\begin{array}{c} x_{k}-x^{*}=P_{Sp^{⊥}\left(A_{k}^{T}\right)}P_{Sp^{⊥}\left(A_{k-1}^{T}\right)}\cdots P_{Sp^{⊥}(A_{1}^{T})}\left(x_{0}-x^{*}\right). \end{array}$$

Note that if *A* ∈ *ℝ* ^*M*×*N*^ is a full column rank with *M* < *N*, then the simple block Kaczmarz update is as follows:

(6) xj+1=xj+Ai†bi−Aixj=xj+Ai†Aix∗−xji=jmod⁡k+1,

where *A*_*i*_^†^ is the pseudo-inverse of *A*_*i*_ and *A*_*i*_^†^ *A*_*i*_ is the orthogonal projection onto *Sp*(*A*_*i*_^*T*^). Then, we get the same equation as (3), and subsequently we get (5),

(7) $$\begin{array}{c} x_{j+1}-x^{*}=x_{j}-x^{*}-P_{Sp\left(A_{i}^{T}\right)}\left(x_{j}-x^{*}\right). \end{array}$$

### 2.2. Exponential Convergence

**Theorem 1 .**

Let **x** ^*^ be the solution of consistent *A* **x** = **b**, where *A* ∈ *ℝ* ^*M*×*M*^ is full rank. Let *A* be row-partitioned as {*A*_1_,…, *A*_*k*_} where *A*_*i*_ ∈ *ℝ* ^*M*_*i*_×*M*^ and *M*_1_ + *M*_2_,…, +*M*_*k*_ = *M*. Then, the simple block Kaczmarz converges exponentially and the convergence rate depends of the number of blocks.

**Proof**

By (3) and orthogonal projection,

(8) $$\begin{array}{c} {\left\|x_{j+1}-x^{*}\right\|}_{2}^{2}={\left\|x_{j}-x^{*}\right\|}_{2}^{2}-{\left\|P_{Sp(A_{i}^{T})}(x_{j}-x^{*})\right\|}_{2}^{2}. \end{array}$$

So,

(9) $$\begin{array}{c} {\left\|x_{j+1}-x^{*}\right\|}_{2}^{2}\leq {\left\|x_{j}-x^{*}\right\|}_{2}^{2}; \end{array}$$

**x**_*j*_ − **x** ^*^ depends on the initial condition ${\tilde{x}}_{0}=x_{0}-x^{*}$, and this dependence is scale-invariant. To see this, let **e**_*j*_ = **x**_*j*_ − **x** ^*^ and consider $c{\tilde{x}}_{0}$ where *c* ∈ *ℝ*. By (4),

(10) ej+1cx~0=PSp⊥Aj+1Tejcx~0=PSp⊥Aj+1TPSp⊥AjT⋯PSp⊥A1Te0cx~0=PSp⊥Aj+1TPSp⊥AjT⋯PSp⊥A1Tcx~0=cPSp⊥Aj+1TPSp⊥AjT⋯PSp⊥A1Te0x~0=cej+1(x~0).

We will first show that if **x**_0_ ≠ **x** ^*^, then ‖**x**_*k*_ − **x** ^*^‖_2_ < ‖**x**_0_ − **x** ^*^‖_2_. By the way of contradiction, assume that **x**_0_ ≠ **x** ^*^ and ‖**x**_*k*_ − **x** ^*^‖_2_ = ‖**x**_0_ − **x** ^*^‖_2_. By (9),

(11) $$\begin{array}{c} {\left\|x_{k}-x^{*}\right\|}_{2}\leq {\left\|x_{k-1}-x^{*}\right\|}_{2}\cdots <{\left\|x_{0}-x^{*}\right\|}_{2} \end{array}$$

and therefore ‖**x**_*l*_ − **x** ^*^‖_2_ = ‖**x**_0_ − **x** ^*^‖_2_ for all 1 ≤ *l* ≤ *k*. By (3), *P*_*Sp*(*A*_*l*_^*T*^)_(**x**_*l*−1_ − **x** ^*^) = 0 for all 1 ≤ *l* ≤ *k*. By (8), we get **x**_*l*_ = **x**_0_ for all 1 ≤ *l* ≤ *k*. This implies that *P*_*Sp*(*A*_*l*_^*T*^)_(**x**_0_ − **x** ^*^) = 0 for all 1 ≤ *l* ≤ *k*. So,

(12) PSp⊥AkT∩Sp⊥AkT⋯∩Sp⊥A1Tx0−x∗=0,PSp⊥(AT)(x0−x∗)=0.

Since *A* is full column rank, we get **x**_0_ = **x** ^*^, which is a contradiction. So we know that ‖**x**_*k*_ − **x** ^*^‖_2_ < ‖**x**_0_ − **x** ^*^‖_2_ (for one full cycle of *k*-iterations).

By compactness, there exists an *ϵ* ∈ (0,1) such that, for all ${\tilde{x}}_{0}=x_{0}-x^{*}\in S^{N-1}$,

(13) $$\begin{array}{c} {\left\|x_{k}-x^{*}\right\|}_{2}\leq 1-\epsilon . \end{array}$$

By (10) and (13),

(14) xk−x∗2=x~02ekx~0x~02≤1−ϵx~02,xk−x∗2≤(1−ϵ)x0−x∗2.

Now consider iteration for *q* cycles:

(15) xqk−x∗2≤1−ϵqx0−x∗2,xqk−x∗2≤1−ϵ1/kqkx0−x∗2.

Therefore, we conclude that the exponential decay depends on the number of blocks *k*. Note that *k* = *M* for regular simple Kaczmarz and the exponential decay depends on the number of rows in this case. The randomized Kaczmarz algorithm proposed by Strohmer and Vershynin [4] avoids this and it converges in expectation as *𝔼*‖**x**_*p*_ − **x** ^*^‖_2_^2^ ≤ (1 − *κ*(*A*)^−2^)^*p*^‖**x**_0_ − **x** ^*^‖_2_^2^, where *κ*(*A*) = ‖*A*‖_*F*_‖*A* ^†^‖_2_ is the scaled condition number of matrix *A* with *A* ^†^ is the pseudoinverse of *A*.

### 2.3. Iterative Subspace Projection Approach

We can use the following theorem (in [9, 11]) to show the convergence of regular block Kaczmarz method.

**Theorem 2 .**

Let *M*_1_, *M*_2_,…, *M*_*k*_ be closed subspaces of the real Hilbert space *ℍ*. Let *M* = ∩_*i*=1_^*k*^ *M*_*i*_ and *P*_*M*_*i*__  (*i* = 1,…, *k*) be orthogonal projection on *M*_*i*_. Then, for each **x** ∈ *ℍ*,

(16) $$\begin{array}{c} \underset{q\to \infty }{\lim}{\left(P_{M_{k}}P_{M_{k-1}}\cdots P_{M_{1}}\right)}^{q}x=P_{M}x, \end{array}$$

where *P*_*M*_ is the orthogonal intersection projection.

The block Kaczmarz is an alternating projection method with *M*_1_ = *Sp* ^⊥^(*A*_1_^*T*^),…, *M*_*k*_ = *Sp* ^⊥^(*A*_*k*_^*T*^). Also,

(17) $$\begin{array}{c} P_{M_{1}}=P_{Sp^{⊥}(A_{1}^{T})},\ldots ,P_{M_{k}=Sp^{⊥}\left(A_{k}^{T}\right)},\\ M=Sp^{⊥}\left(A_{1}^{T}\right)\cap \cdots \cap Sp^{⊥}\left(A_{k}^{T}\right)=Sp^{⊥}\left(A^{T}\right). \end{array}$$

Since *A* is full column rank, *Sp* ^⊥^(*A* ^*T*^) = {0} and *P*_*M*_ = {0}. After *q* cycles,

(18) $$\begin{array}{c} x_{qk}-x^{*}={\left(P_{M_{k}}P_{M_{k-1}}\cdots P_{M_{1}}\right)}^{q}\left(x_{0}-x*\right). \end{array}$$

By Theorem 2, lim⁡_*q*→*∞*_ **x**_*qk*_ − **x** ^*^ = 0 and lim⁡_*q*→*∞*_ **x**_*qk*_ = **x** ^*^. Galántai in [9] gives a bound for ‖**x**_*qk*_ − **x** ^*^‖_2_ in terms of principle angles between *M*_*i*_'s.

### 2.4. Bound for Block Kaczmarz in Terms of Principle Angles

Smith et al. established the following convergence theorem for applying the alternating projection method in tomography [9, 13].

**Theorem 3 .**

Let *M*_1_, *M*_2_,…, *M*_*k*_ be closed subspaces of the real Hilbert space *ℍ*. Let *M* = ∩_*i*=1_^*k*^ *M*_*i*_ and *P*_*M*_*i*__  (*i* = 1,…, *k*) be orthogonal projection on *M*_*i*_ (*P*_*M*_ is the orthogonal intersection projection). Let *θ*_*j*_ = *α*(*M*_*j*_, ∩_*i*=*j*+1_^*k*^ *M*_*i*_); then, for each **x** ∈ *ℍ* and integer *q* ≥ 1,

(19) PMkPMk−1⋯PM1qx−PMx22  ≤1−Πj=1k−1sin2θjqx−PMx22,

where *P*_*M*_ is the orthogonal intersection projection.

In the special case of the block Kaczmarz, we have *ℍ* = *ℝ* ^*N*^, *M*_1_ = *Sp* ^⊥^(*A*_1_^*T*^),…, *M*_*k*_ = *Sp* ^⊥^(*A*_*k*_^*T*^). Also, *P*_*M*_1__ = *P*_*Sp*^⊥^(*A*_1_^*T*^)_,…, *P*_*M*_*k*__ = *P*_*Sp*^⊥^(*A*_*k*_^*T*^)_ and *M* = *Sp* ^⊥^(*A*_1_^*T*^)∩⋯∩*Sp* ^⊥^(*A*_*k*_^*T*^) = *Sp* ^⊥^(*A* ^*T*^). Since *A* is full column rank, *Sp* ^⊥^(*A* ^*T*^) = {0} and *P*_*M*_ = {0}. Therefore, after *q* cycles,

(20) xqk−x∗22=PMkPMk−1⋯PM1q(x0−x∗)22≤1−Πj=1k−1sin2θjqxo−x∗22,

where *θ*_*j*_ is as defined in Theorem 3. Note that the exponential decay rate depends on the number of blocks *k* as shown below:

(21) $$\begin{array}{c} {\left\|x_{qk}-x^{*}\right\|}_{2}^{2}\leq {\left[{\left(1-\Pi_{j=1}^{k-1}\sin^{2}\theta_{j}\right)}^{1/k}\right]}^{qk}{\left\|x_{o}-x^{*}\right\|}_{2}^{2}. \end{array}$$

Galántai in [9] developed another bound (for *A* ∈ *ℝ* ^*M*×*M*^) by defining a new matrix *X*_*i*_ for each block *A*_*i*_ as follows.

**Theorem 4 .**

Let **x** ^*^ be the solution of *A* **x** = **b** for a consistent linear system with *A* ∈ *ℝ* ^*M*×*M*^. Let *A* be row-partitioned as {*A*_1_,…, *A*_*k*_} where *A*_*i*_ ∈ *ℝ* ^*M*_*i*_×*N*^. Let *M*_1_ = *Sp* ^⊥^(*A*_1_^*T*^),…, *M*_*k*_ = *Sp* ^⊥^(*A*_*k*_^*T*^) and *A*_*i*_ *A*_*i*_^*T*^ = *LL* ^*T*^ be the Cholesky decomposition of *A*_*i*_ *A*_*i*_^*T*^. Define *X*_*i*_ = *A*_*i*_^*T*^ *L* ^−*T*^ and *X* = [*X*_1_,…, *X*_*k*_]. Then, for each **x** ∈ *ℝ* ^*N*^ and integer *q* ≥ 1,

(22) xqk−x∗22≤1−det⁡XTXqxo−x∗22=1−det⁡XTX1/kqkxo−x∗22.

### 2.5. Special Case: Simple Kaczmarz for *A* ∈ *ℝ* ^*M*×*M*^

Note that this section assumes that *A* ∈ *ℝ* ^*M*×*M*^. The block Kaczmarz algorithm is equivalent to the simple Kaczmarz algorithm if the number of blocks *k* is equal to the number of rows *M*. In this case, *A*_*i*_ *A*_*i*_^*T*^ = ‖**a**_*i*_‖_2_^2^ = *LL* ^*T*^. Therefore, *L* = ‖**a**_*i*_‖_2_ and *L* ^−*T*^ = 1/‖**a**_*i*_‖_2_. This implies that *X*_*i*_ = [**a**_*i*_/‖**a**_*i*_‖_2_]. Then, *X* ∈ *ℝ* ^*M*×*M*^ is defined as

(23) $$\begin{array}{c} X=\left[\frac{a_{1}}{{\left\|a_{1}\right\|}_{2}},\ldots ,\frac{a_{M}}{{\left\|a_{M}\right\|}_{2}}\right]. \end{array}$$

Assume the matrix *A* has normalized rows and we pick a row at each iteration uniformly randomly. Note that this assumption is feasible as scaling a row of *A* and the corresponding measurement in **b** does not change the solution **x**.

*X* is the Gram matrix with 0 ≤ det⁡(*X* ^*T*^ *X*) ≤ ‖**x**_1_‖_2_^2^‖**x**_2_‖_2_^2^ ⋯ ‖**x**_*M*_‖_2_^2^. Since ‖**x**_*i*_‖_2_ = 1 and *X* is full rank, we have 0 < det⁡(*X* ^*T*^ *X*) ≤ 1. Using Theorem 4, we get the following deterministic bound:

(24) $$\begin{array}{c} {\left\|x_{qM}-x^{*}\right\|}_{2}^{2}\leq {\left[{\left(1-\det\left(X^{T}X\right)\right)}^{1/M}\right]}^{qM}{\left\|x_{0}-x^{*}\right\|}_{2}^{2}. \end{array}$$

Since *A* is normalized, we get *X* = *A* ^*T*^ and therefore

(25) $$\begin{array}{c} {\left\|x_{qM}-x^{*}\right\|}_{2}^{2}\leq {\left[{\left(1-\det\left(AA^{T}\right)\right)}^{1/M}\right]}^{qM}{\left\|x_{0}-x^{*}\right\|}_{2}^{2}. \end{array}$$

Bai and Liu (in [14]) uses the Meany Inequality to develop a general form of this inequality.

### 2.6. Randomized Kaczmarz Method

Several methods of randomized Kaczmarz are discussed in this section.

### 2.7. Randomization Based on Row *ℓ*_2_ Norms Method

Strohmer and Vershynin (in [4]) developed a randomized Kaczmarz algorithm that picks a row of *A* in a random fashion with probability proportional with *ℓ*_2_ norm of that row. They proved that this method has exponential expected convergence rate. Since the rows are picked based on a probability distribution generated by the *ℓ*_2_ norms of the rows of *A*, it is clear that scaling some of the equations does not change the solution set. However, it may drastically change the order of the rows picked at each iteration. Censor et al. discuss (in [15]) that this should not be better than the simple Kaczmarz as picking a row based on its *ℓ*_2_ norm does not change the geometry of the problem.  Theorem 5 is from [4].

**Theorem 5 .**

Let **x** ^*^ be the solution of *A* **x** = **b** Then, Algorithm 1 converges to **x** ^*^ in expectation, with the average error

(26) $$\begin{array}{c} E{\left\|x_{p}-x^{*}\right\|}_{2}^{2}\leq {\left(1-\kappa {\left(A\right)}^{-2}\right)}^{p}{\left\|x_{0}-x^{*}\right\|}_{2}^{2}, \end{array}$$

where *κ*(*A*) = ‖*A*‖_*F*_‖*A* ^†^‖_2_ is the scaled condition number of matrix *A* with *A* ^†^ is the left pseudoinverse of *A*.

Note that *A* is a full column matrix (*A* ∈ *ℝ* ^*M*×*N*^ with rank⁡(*A*) = *N*) and therefore we define *A* ^†^ as left pseudoinverse of *A*. We observe that the randomization should work better than the simple (cyclic) Kaczmarz algorithm for matrices with highly coherent rows (e.g., matrices generated by the computerized tomography). Since the Kaczmarz algorithm is based on projections, the convergence will be slow if the consecutive rows selected are highly coherent (i.e., the angle between **a**_*i*_ and **a**_*i*+1_ is small). Picking rows randomly (not necessarily based on the *ℓ*_2_ norms) makes picking more incoherent rows possible in each iteration. Therefore, the randomization may be useful for certain applications such as medical imaging. Note that matrix *A* generated by computerized tomography has coherent and sparse rows due to physical nature of data collection. In fact, using Theorem 5, we can develop the following proposition.

**Proposition 6 .**

Let *A* **x** = **b** be a consistent linear system of equations (*A* ∈ *ℝ* ^*M*×*N*^) and let **x**_0_ be an arbitrary initial approximation to the solution of *A* **x** = **b**. For *k* = 1,2,…, compute

(27) $$\begin{array}{c} x_{p+1}=x_{p}+\frac{b_{r(i)}-\langle a_{r\left(i\right)},x_{p}\rangle }{{\left\|a_{r(i)}\right\|}_{2}^{2}}a_{r(i)}, \end{array}$$

where *r*(*i*) is chosen from the set {1,2,…, *M*} at random, with “any probability distribution.” Let **x** ^*^ be the solution of *A* **x** = **b**. Then,

(28) $$\begin{array}{c} E{\left\|x_{p}-x^{*}\right\|}_{2}^{2}\leq {\left(1-\kappa {\left(B\right)}^{-2}\right)}^{p}{\left\|x_{0}-x^{*}\right\|}_{2}^{2}, \end{array}$$

where *κ*(*B*) = ‖*B*‖_*F*_‖*B* ^†^‖_2_ is the scaled condition number of a matrix *B* that is obtained by some row-scaling of *A*.

**Proof**

This is due to the fact that row-scaling of *A* (with scaling of the corresponding *b*) does not change the geometry of the problem and we can scale the rows to generate any probability distribution. In other words, we can obtain another matrix *B* from *A* by scaling its rows in such a way that picking the rows of *B* based on the *ℓ*_2_ norms of the rows will be equivalent to picking the rows of *A* based on the chosen probability distribution. Therefore, clearly, any randomization of the row selection will have exponential convergence; however, the rate will depend on the condition number of another matrix. For example, if we use uniform distribution, we can then normalize each row to have matrix *B* as follows and then pick the rows at random with probability proportional to the norms of the rows:

(29) $$\begin{array}{c} B={\left[\frac{a_{1}}{{\left\|a_{1}\right\|}_{2}},\ldots ,\frac{a_{M}}{{\left\|a_{M}\right\|}_{2}}\right]}^{T}. \end{array}$$

### 2.8. Randomization Based on Subspace Angles Method

Our approach iterates through the rows of *A* based on a probability distribution using the hyperplane (subspace) angles. Therefore, it is immune to scaling or normalization. This approach first generates a probability distribution based on the central angle(s) {*θ*_*i*,*j*_} between the hyperplanes (represented by the rows of *A* **x** = **b**). Then, it randomly picks two hyperplanes using this probability distribution. This is followed by a two-step projection on these hyperplanes (see Algorithm 2).

### 2.9. *P*-Subspaces Method

A new method has been developed which is intended to better accommodate the coherency of nonorthogonal data measurements. This next section makes contributions towards proving the statistical convergence of the randomized Kaczmarz orthogonal subspace (RKOS) algorithm. As described in [16], the RKOS initially uses *ℓ* ^2^-norm random hyperplane selection and subsequent projection into a constructed *P*-dimensional orthogonal subspace *S*_*P*_ comprised of an additional *P* − 1 hyperplanes selected uniformly at random.

Algorithm 3 uses a recursive method to solve for the projections into the orthogonal subspace which is constructed using Gram-Schmidt (GS) procedure. However, a second approach demonstrates an alternate method of arriving at similar results, based upon an a closed form matrix for QR decomposition [17] of projection blocks.

In each of the above cases, vector operations inside the orthogonal subspace preserve the *ℓ* ^2^-norm and reduce errors that would normally be induced for coherent nonorthogonal projections which may be present in the simple Kaczmarz.

#### 2.9.1. Orthogonal Subspaces

A statistical convergence analysis for Randomized Kaczmarz Orthogonal Subspace (RKOS) method is developed assuming identically and independently distributed (IID) random variables as vector components of each row of the measurement matrix *A*.

*(a) Orthogonal Construction.* In many problems, *M* ≫ *N* and fast but optimal solutions are needed, often in noisy environments. In most cases, orthogonal data projection sampling is not feasible due to the constraints of the measurement system. The algorithm and procedure for the RKOS method are given in reference [16] and are intended to construct orthogonal measurements subspaces (see Algorithm 3).

The general technique is to solve using a constructed orthogonal basis from a full rank set of linearly independent measurements in for each subspace in Gram-Schmidt fashion [18, 19].

The subspace estimation may be computed as *P*-dimensional subspace projection into the subspace orthonormal vector basis:

(30) $$\begin{array}{c} x_{S_{P}}=\overset{P}{\underset{l=1}{\sum }}\langle {\hat{u}}_{l},x\rangle {\hat{u}}_{l}, \end{array}$$

where **x**_*S*_*P*__ in *S*_*P*_⊆*S*_*N*_ subspace is the *P*-dimensional solution approximation which becomes exact for *S*_*P*=*N*_ for **x**_*S*_*P*=*N*__ ∈ *ℝ* ^*N*^ in the noiseless, self-consistent, case (the *u* vector with the hat symbol $\hat{u}$ indicates unit *ℓ* ^2^-norm).

*(b) Modified Kaczmarz.* The standard Kaczmarz equation is essentially iterative projections into a single subspace of dimension one; based upon the sampling hyperplanes, these projections are often oblique, especially in highly-coherent sampling.

The approach herein is motivated towards constructing an iterative algorithm based upon Kaczmarz which may be accelerated while controlling the potential projection errors and incurring reasonable computational penalty. The algorithm is simply to add subspaces of larger dimensions. Let

(31) $$\begin{array}{c} x-x_{k+1}=x-x_{k}-\overset{P}{\underset{l=1}{\sum }}\langle {\hat{u}}_{l},x-x_{k}\rangle {\hat{u}}_{l}. \end{array}$$

It is convenient to make a substitution as follows:

(32) $$\begin{array}{c} z_{k+1}=x-x_{k+1}. \end{array}$$

Using above substitution and orthonormal condition (It is worthwhile to note that in the problem setup, a fixed vector is projected into a randomized *P*-dimensional subspace, where algebraic orthogonality was used to obtain (34). In the this statistical treatment of the same equation, the expectation of two random unit vectors vanishes for independent uncorrelated zero mean probability distribution functions, providing the statistical orthogonality on average satisfying (34).) $\langle {\hat{u}}_{j},{\hat{u}}_{k}\rangle =\delta_{j,k}$, where the Kronecker

(33) $$\begin{array}{c} \delta_{j,k}=\left\{\begin{array}{cc} 0, & \text{if}\;\;j\neq k,\\ 1, & \text{if}\;\;j=k, \end{array}\right. \end{array}$$

find the *ℓ* ^2^-norm squared of **z**_*k*+1_:

(34) $$\begin{array}{c} {\left\|z_{k+1}\right\|}_{2}^{2}={\left\|z_{k}\right\|}_{2}^{2}-\overset{P}{\underset{l=1}{\sum }}{\left|\langle {\hat{u}}_{l},z_{k}\rangle \right|}^{2}. \end{array}$$

The ensemble average of the above equation (34) yields the convergence result, which is the main topic of this section.

#### 2.9.2. Convergence for IID Measurement Matrix

Firstly, the expectation of a single random projection is computed. In the second step, the terms are summed for the *P*-dimensional subspace. Experimental results are included in a latter section.

*(a) Expectation of IID Projections.* Consider the expectation of the *ℓ* ^2^-norm squared of the projection of fixed vector **x** ∈ *ℝ* ^*N*×1^ onto a random subspace basis *U*_*P*_∈ of dimension *P*:

(35) $$\begin{array}{c} E\left[{\left\|U_{P}^{T}x\right\|}_{2}^{2}\right], \end{array}$$

where the matrix basis *U*_*P*_ ∈ *ℝ* ^*NxP*^ is comprised of *P*-columns of unit vectors ${\hat{u}}_{j}\in {\mathbb{R}}^{N}$ in a constructed orthogonal basis for

(36) u^j⟶U^j=Uj,1,…,Uj,N1Cσ=UjUj22 ∀j∈1,…,P,

where the upper case components *U*_*j*,*i*_ represent the (*j*, *i*)th IID random variable component, and normalization constant *C*_*σ*_ is to be determined.

Further noting that complex conjugate (·)^*^ reduces to transpose (·)^*T*^ for real components, the *ℓ* ^2^-norm squared of the projection expands to

(37) $$\begin{array}{c} {\left\|U_{P}^{T}x\right\|}_{2}^{2}=x^{T}U_{P}U_{P}^{T}x. \end{array}$$

In the next section, the goal is to find the expected value for outer product of the projection:

(38) $$\begin{array}{c} E\left[x^{T}{\hat{U}}_{j}{\hat{U}}_{j}^{T}x\right]\;\forall j\in \left[1,\ldots ,P\right]. \end{array}$$

*(b) Unit Vector.* The deterministic identity for the magnitude of a unit vector is well known result for $\hat{u}\in {\mathbb{R}}^{N}$:

(39) $$\begin{array}{c} {\left\|\hat{u}\right\|}_{2}^{2}=\overset{N}{\underset{i=1}{\sum }}\frac{u_{i}^{2}}{{\left\|u\right\|}_{2}^{2}}=1. \end{array}$$

The following statistical result must apply for the *j*th column unit vector:

(40) EU^j22=EU^jTU^j=1=EUj,12+⋯+Uj,N21Cσ2.

*(c) Normalization of Random Unit Vector.* Denote ${\hat{U}}_{j}$ as the *j*th random variable unit-norm vector associated with a set of column vectors {**U**_*j*_}_*j*∈1,…,*P*_ comprising a random subspace matrix *U*_*N*×*P*_ having IID random variable components *U*_*j*,*i*_. However, no additional assumptions on the distribution of the random variables are made at this time, other than IID. The expectation of both sides of (40) for random vector **U**_*j*_ are found such that

(41) $$\begin{array}{c} E\overset{N}{\underset{i=1}{\sum }}\frac{U_{j,i}^{2}}{C_{\sigma }^{2}}=\overset{N}{\underset{i=1}{\sum }}E\left[\frac{U_{j,i}^{2}}{C_{\sigma }^{2}}\right]=1,\\ N\times \frac{E\left[U_{j,i}^{2}\right]}{C_{\sigma }^{2}}=1. \end{array}$$

Solving above for each unit vector component in this treatment implies a random variable *U*_*j*,*i*_ with zero mean and variance as follows:

(42) $$\begin{array}{c} E\left[U_{j,i}^{2}\right]=\sigma_{j,i}^{2}=\frac{C_{\sigma }^{2}}{N}\;\forall U_{j,i\in 1,\ldots ,N}\in f\left(U_{j,i}\right), \end{array}$$

where *f*(*U*_*i*,*j*_) is the associated IID probability distribution.

*(d) P-Dimensional Random Projection.* The next step is to compute the expectation of the magnitude of the projection of fixed vector **x** onto random *P*-dimensional orthonormal subspace *U*_*P*_ projection term by term. Let **α** ∈ *ℝ* ^*P*^ be a column vector defined as **α** = *U*_*P*_^*T*^ **x** and find the *ℓ* ^2^-norm squared:

(43) α22=α12+α22+⋯+αP2=UPTx22=xTUPUPTx,

where

(44) αj2=u^j,x2=uj,1x1+⋯+uj,NxN2=∑i,kN,Nuj,kuj,ixkxiuj22.

Let upper case *U*_*j*,*k*_ denote the *k*th IID element random (this is not the same *k*-variable as the Kaczmarz iteration variable) variable of the *j*th column vector **U**_*j*_ associated with column vector **u**_**j**_; let **x** vector denote a fixed point. Next, take the expectation of the term over the possible outcomes of *U*_*j*,*k*_ random variables. Using the IID assumption, the expected value for a single projection component preserves terms squared as follows:

(45) Eαj2=E∑i,kN,NUj,kUj,ixkxiCσ2=∑i,kN,NEUj,kUj,ixkxiCσ2=∑k=1NEUk2xk2Cσ2=∑kNEUj,k2Cσ2xk2=EUj,k2Cσ2∑kNxk2=EUj,k2Cσ2x22=1Cσ2Cσ2Nx22=1Nx22.

It is now possible to determine the expectation for *P*-terms of the projection as

(46) $$\begin{array}{c} E\left[{\left\|\alpha \right\|}_{2}^{2}\right]=E\left[\overset{P}{\underset{j=1}{\sum }}\alpha_{j}^{2}\right]=\frac{P}{N}{\left\|x\right\|}_{2}^{2} \end{array}$$

subject to IID constraint on ${\hat{U}}_{j}$ where it is further noted that *σ* ^2^ *N* = *C*_*σ*_^2^ in (42).

*(e) Error per Iteration.* For a given *k*th Kaczmarz iteration, the expectation of the projection of fixed vector **x** onto the random *P*-dimensional subspace *U*_*P*_ is known from above. The total convergence expectation may then be computed, using a method similar to Strohmer's, starting (recall that derivation of (47) requires orthogonality among the ${\hat{u}}_{l}$ subspace basis vectors) with (47):

(47) $$\begin{array}{c} {\left\|z_{k+1}\right\|}_{2}^{2}={\left\|z_{k}\right\|}_{2}^{2}-\overset{P}{\underset{l=1}{\sum }}{\left|\langle z_{k},{\hat{u}}_{l}\rangle \right|}^{2} \end{array}$$

(48) Ek+1 ∣ z0,z1,…,zkzk+122 =Ek+1 ∣ z0,z1,…,zkzk22−∑l=1Pzk,u^l2 =Ek+1 ∣ z0,z1,…,zkzk22−Ek+1 ∣ z0,z1,…,zk∑l=1Pzk,u^l2.

We identify the term on the right as

(49) Ek+1 ∣ z0,z1,…,zk∑l=1Pzk,u^l2=Ek+1 ∣ z0,z1,…,zkUPzk22=PN×Ek+1 ∣ z0,z1,…,zkzk22.

The results from the two equations ((49) and (48)) above may then be combined to obtain

(50) Ek+1 ∣ z0,z1,…,zkzk+122  =1−PN×Ek ∣ z0,z1,…,zk−1zk22,

where the expectation on the right hand side includes *k* + 1 → *k* accounting for the previous iteration. Next, apply induction to arrive at the expectation for the whole iterative sequence up to the *β*th iteration given that **z**_0_ ≡ **x** − **x**_0_:

(51) $$\begin{array}{c} E_{\left\{\beta +1\;|\;z_{0}\right\}}\left[{\left\|z_{\beta +1}\right\|}_{2}^{2}\right]={\left(1-\frac{P}{N}\right)}^{\beta }{\left\|z_{0}\right\|}_{2}^{2}\;\forall \beta \in 1,2,3,\ldots . \end{array}$$

*(f) Asymptotic Convergence.* The statistical ensemble average of the above equation (34) for the *β*th iteration yields the convergence result given in (51). These results assume random variables identically and independently distributed but compare well to others in the literature, such as the convergence result in Strohmer and Vershynin [20].

The theoretical convergence iterative limit for uniform random IID sampling was compared to numerical simulations using random solution vector point on a unit sphere. Equation (52) has an asymptotic form:

(52) E{β+1|z0}zβ+122z022  =lim⁡β→∞1−PNβ≃e−βPN,P=dim⁡(SP), β≫1,2,3,…⟶k∈P,2P,3P,….

For comparison, recall the convergence for RK method of Strohmer for IID measurements with *R* = *N* which is approximately

(53) $$\begin{array}{c} \frac{E_{\left\{k+1\;|\;z_{0}\right\}}\left[{\left\|z_{k+1}\right\|}_{2}^{2}\right]}{{\left\|z_{0}\right\|}_{2}^{2}}={\left[1-\frac{1}{N}\right]}^{k}\\ \underset{k\to \infty }{\lim}{\left[1-\frac{1}{N}\right]}^{k}≃e^{-k/N}\;\forall k\gg 1,2,3,\ldots . \end{array}$$

Estimated noise bound convergence complexity to *ϵ* error is *O*(*N* ^2^). Since the value of  **z**_0_ is given, the expectation is known to be the same.

**Figure 1:**
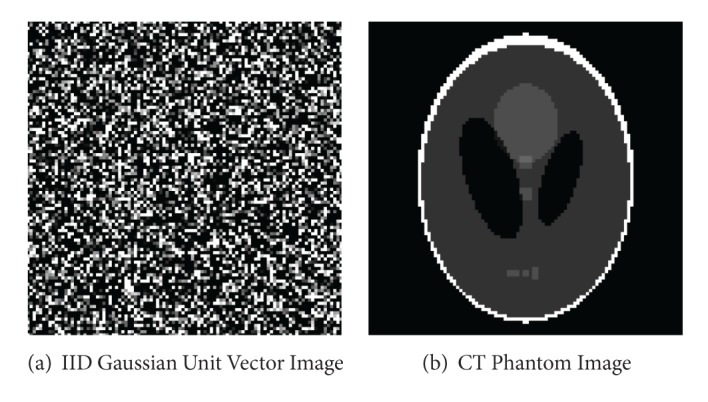
Representative test data.

*(g) Theory and Simulation.* Simulations in [16] compare theory to Gaussian IID with noise variance added to the measurements with magnitude *β* = 0.05 (about five percent) and iteration termination at *β* = 0.05/4 = 0.0125. In the first problem, the exact solution **x** is chosen as a random point on the unit sphere—which is illustrated in Figure 1(a). In a second problem, a measurement of the standard phantom using parallel beam measurements is included, which contains coherent measurements.

### 2.10. Regular Versus Randomized Kaczmarz Method

The randomized Kaczmarz's algorithm developed by Strohmer and Vershynin in [4] has the following convergence in expectation:

(54) $$\begin{array}{c} E{\left\|x_{qM}-x^{*}\right\|}_{2}^{2}\leq {\left(1-\frac{1}{\kappa {\left(A\right)}^{2}}\right)}^{qM}{\left\|x_{0}-x^{*}\right\|}_{2}^{2}, \end{array}$$

where *κ*(*A*) = ‖*A*‖_*F*_‖*A* ^†^‖_2_ is the scaled condition number of matrix *A* with *A* ^†^ as the left pseudoinverse of *A*. The bound for regular Kaczmarz is given in (25). Note that we assume *A* ∈ *ℝ* ^*M*×*M*^. Now, we need to compare (1 − (1/‖*A*‖_*F*_^2^‖*A* ^†^‖_2_^2^)) and (1 − det⁡(*AA* ^*T*^))^1/*M*^ to assess which bound is tighter. Let *σ*_1_ ≥ *σ*_2_ ≥ ⋯≥*σ*_*M*_ > 0 be ordered singular values of *A*. Then,

(55) $$\begin{array}{c} {\left\|A^{†}\right\|}_{2}^{2}=\frac{1}{\sigma_{N}^{2}},\\ {\left\|A\right\|}_{F}^{2}=\overset{M}{\underset{i=1}{\sum }}\sigma_{i}^{2}. \end{array}$$

Also, note that

(56) $$\begin{array}{c} AA^{T}=\left[\begin{array}{cccc} 1 & \cos\theta_{12} & \cdots & \cos\theta_{1M}\\ \cos\theta_{21} & 1 & \cdots & \cos\theta_{2M}\\ \vdots & \vdots & \cdots & \vdots \\ \cos\theta_{M1} & \cos\theta_{M2} & \cdots & 1 \end{array}\right], \end{array}$$

where *θ*_*ij*_ denotes the angles between the rows *a*_*i*_ and *a*_*j*_ of *A*. Then,

(57) $$\begin{array}{c} \det\left(AA^{T}\right)\leq \overset{M}{\underset{i=1}{\prod }}\overset{M}{\underset{j=1}{\sum }}\cos^{2}\theta_{ij}. \end{array}$$

Note that

(58) $$\begin{array}{c} \overset{M}{\underset{i=1}{\prod }}\sigma_{i}^{2}\left(A\right)=\overset{M}{\underset{i=1}{\prod }}\lambda_{i}\left(A^{T}A\right)=\det\left(A^{T}A\right)=\det\left(AA^{T}\right); \end{array}$$

therefore,

(59) $$\begin{array}{c} {\left[1-\det\left(AA^{T}\right)\right]}^{1/M}={\left(1-\overset{M}{\underset{i=1}{\prod }}\sigma_{i}^{2}\right)}^{1/M}. \end{array}$$

Now, (54) and (25) become

(60) $$\begin{array}{c} E{\left\|x_{qM}-x^{*}\right\|}_{2}^{2}\leq {\left(1-\frac{\sigma_{M}^{2}}{\sum_{i=1}^{M}\sigma_{i}^{2}}\right)}^{qM}{\left\|x_{0}-x^{*}\right\|}_{2}^{2},\\ {\left\|x_{qM}-x^{*}\right\|}_{2}^{2}\leq {\left[{\left(1-\overset{M}{\underset{i=1}{\prod }}\sigma_{i}^{2}\right)}^{1/M}\right]}^{qM}{\left\|x_{0}-x^{*}\right\|}_{2}^{2}. \end{array}$$

### 2.11. Complexity Measurement Methods

In order to visualize and assess the relative performance of the well-known common methods of Kaczmarz, a simple routine was written in Octave [21] to record the total central processing unit (CPU) times for each method and variable matrix sizes of interest. The Linux (tm) kernel was modified to include the real-time patches for the x86/64 CPU architecture, and the process was run on a (see the* cset* in the cpuset package) shielded CPU set to single processor unit CPU7 to avoid process contention and interrupts. All system functions were locked to other CPU instances and not allowed to execute on CPU7.

Execution times were measured with* rusage()* calls before and after calls to each method in Octave. The elapsed time has microsecond resolution from the* rusage()* function. In addition, the interrupt balance kernel function was disabled. The status of the CPU cores and interrupts may be observed in  /proc/interrupts. The CPU clock frequency was locked to a fixed value of 800 Mhz for the x86_64 system. Virtual memory was dropped prior to execution to ensure maximum physical memory availability.

In addition to the 64-bit platform, an embedded 32-bit ARM architecture was also configured for computational reference. Due to time constraints, no real-time kernel was implemented for the ARM. The CPU clock frequency was locked to a fixed value of 840 Mhz for the embedded ARM system.

In both of the above benchmark cases, all noncritical network and file system processes and daemons were stopped prior to code execution. In both cases, program execution was monitored and noted to reside in virtual (nonswaped) memory mapped to physical memory.

#### 2.11.1. Methods

The* kaczmarz()*,* randkaczmarz()*, and* cimmino()* methods were called directly from the AIR toolkit [22]. The functions for the block method* chenko()* were implemented from Vasil'chenko and Svetlakov [23] of the form

(61) $$\begin{array}{c} x_{j+1}=x_{j}+A_{i}^{T}{\left(A_{i}A_{i}^{T}\right)}^{-1}\left(b_{i}-A_{i}x_{j}\right)\;i=j\left(\mathrm{mod}k\right)+1 \end{array}$$

for *A*_*i*_ = *E*_*i*_^*T*^ *A*.

The* rkos()* was implemented from [16] algorithm. The function for least squares* ls()* was computed from the equation

(62) $$\begin{array}{c} x^{*}={\left(A^{T}A\right)}^{-1}A^{T}b. \end{array}$$

The function for singular value decomposition* svd()* used the decomposition of the sampling matrix *A* = *U*Σ*V* ^*T*^, and the solution was computed as follows:

(63) $$\begin{array}{c} x^{*}=V\Sigma^{-1}U^{T}Ax^{*}=V\Sigma^{-1}U^{T}b. \end{array}$$

The resulting plots are intended to illustrate how the methods scale with increasing measurement matrix size and not directly used in absolute terms, since each method has various dependencies in hardware and software.

A simple baseline Kaczmarz code was written to ensure a baseline reference was available. The Octave source code for a typical method is included in the inset Listing 1.

#### 2.11.2. Notes Regarding Simulations

The sizes of the matrix blocks equal matrix dimensions until constant block size of sixteen rows. The legend key is as follows:

(1) least squares:* LS()*,
(2) singular value decomposition:* SVD()*,
(3) *Kaczmarz()*,
(4) randomized Kaczmarz:* RK()*,
(5) block method randomized Kaczmarz orthogonal subspaces:* RKOS()*,
(6) *Cimmino()*,
(7) block method* Vasilchencko()*,
(8) reference P1: simple matrix multiply with known *O*(*N* ^2^) complexity,
(9) reference P2: *N* times P1 for known *O*(*N* ^3^) complexity,
(10) reference Kaczmarz as shown in Listing 1.

## 3. Results and Discussion

Here, we compare our angle-based randomization with norm-based randomization of Strohmer and Vershynin [4] in the context of uniform and nonuniform measurement methods. In particular, Shepp-Logan and* sinc2d()* phantom images were used as the solutions in four different simulation experiments [3].  Figure 3(d) shows that our approach (angle-based randomization) provides a better convergence rate over the randomized Kaczmarz (norm-based randomization) in the case of fan-beam sampling of the* sinc2d()* phantom. However, our method is computationally more complex, and therefore we devised the *P*-subspace algorithm (presented in the previous section).

In the experiments which follow, three factors of interest are the sampling angular distribution of the measurements (affects coherence), the algorithms' method of iteration through the sampling hyperplanes, and the rate of convergence of the solution. The simulations were computed in parallel for each of the methods: Kaczmarz (K), randomized Kaczmarz hyperplane angles (RKHA), and randomized Kaczmarz (RK). Iteration is terminated at stopping point defined as the condition when at least one of the three methods attains a normalized error of 10% or less. Estimates for the signal to noise ratio are computed at same common stopping point.

### 3.1. Sampling Distributions

The following experiments compare Kaczmarz (K), randomized Kaczmarz (RK), and randomized Kaczmarz hyperplane angles (RKHA) via simulations. The objectives are to illustrate the effect of row randomization upon the convergence and observe the dependency upon the sampling methods.

#### 3.1.1. Angular Distribution of Sampling Hyperplanes

**Figure 2:**
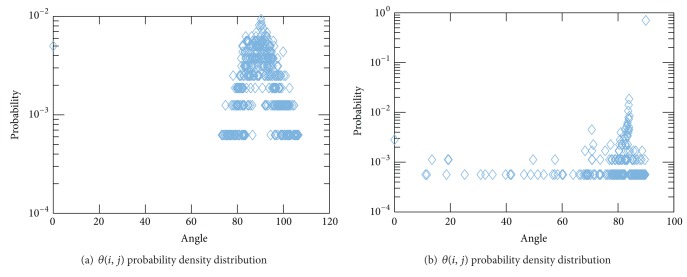
Example angles distribution (*y*-axis) from *AA* ^*T*^ where $\theta_{i,j}=\cos^{-1}(\langle {\hat{a}}_{i},{\hat{a}}_{j}\rangle )$  ∀*i*, *j* ∈ {1,…, *M*} versus angles (*x*-axis) degrees using (a) random and (b) fan-beam data acquisition strategy where the unit norm vectors ${\hat{a}}_{i}$, ${\hat{a}}_{j}$ are selected rows of matrix *A*.

A comparison of the distribution of hyperplane sampling angles in computed tomography (CT) was performed to investigate the convergence rate versus measurement strategy. Example results are presented for iterative convergence of methods K, RK, and RKHA under conditions of random, fan, and parallel beam sampling strategies using the Shepp-Logan phantom (see Figure 1(b)) [22],* paralleltomo.m* and* fanbeamtomo.m* from the AIRtools distribution [22] and* randn()* from the built-in function method [24]. The typical results for the angular distributions are provided in Figures 2(a) and 2(b) and discussed in more detail below.

#### 3.1.2. Sampling Coherence

In linear algebra, the coherence or mutual coherence [25] of a row measurement matrix *A* is defined as the maximum absolute value of the cross-correlations between the normalized rows of *A*.

Formally, let {**a**_1_,…, **a**_*M*_} ∈ *ℝ* ^*N*^ be the set of row vectors of the matrix *A* ∈ *ℝ* ^*M*×*N*^ normalized such that 〈**a**_*i*_, **a**_*i*_〉 = **a**_*i*_^*H*^ **a**_*i*_ = 1 where (·)^*H*^ is the Hermitian conjugate and where *M* > *N*. Let the mutual coherence of *A* be defined as

(64) $$\begin{array}{c} \phi_{i,j}=\underset{1\leq i\neq j\leq M}{\max}\left|a_{i}^{H}a_{j}\right|. \end{array}$$

A lower bound was derived as *ϕ* ≥ (*M* − *N*)/*N*(*M* − 1) in Welch [26].

In the distributions of Figures 2(a) and 2(b), it should be noted that the random sampling is concentrated near 90 degrees probability but fan sampling is less concentrated across the interval [0,90] degrees.

#### 3.1.3. Observations on Effects of Sampling Distribution, Algorithm, and Convergence

**Figure 3:**
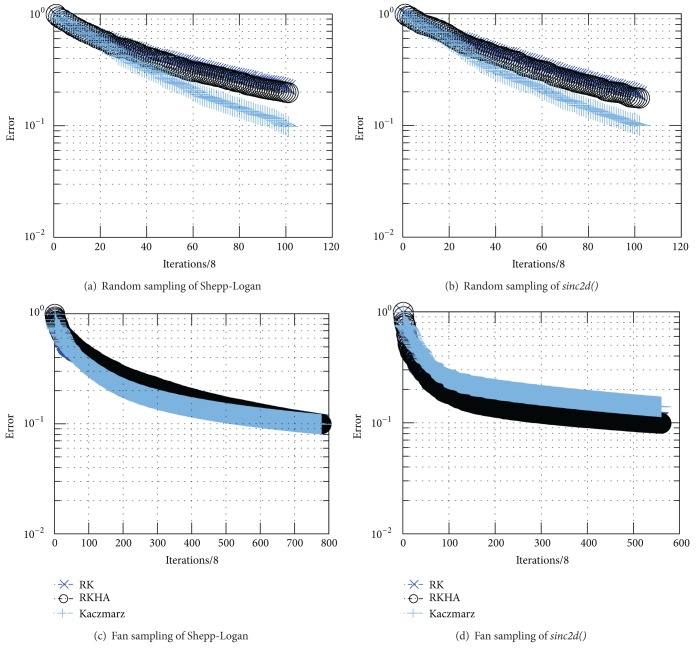
Semilog (*y*-axis) plot example of normalized convergence error results for K, RK, and RKHA on Shepp-Logan phantom using random and fan tomographic data acquisition sampling and stopping at 10 percent error.

Firstly, the convergence rates of K, RK, and RKHA are noted to be closely correlated for the case of random data sampling of the phantom in Figures 3(a) and 3(b). This is consistent with the mean values of coherence near zero for random sampling.

**Figure 4:**
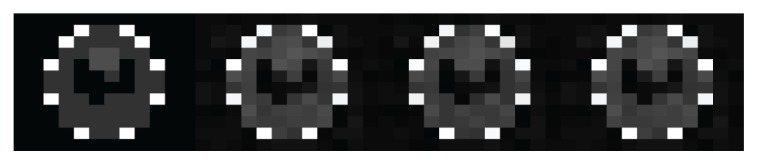
Reconstructed test images for Shepp-Logan with fan sampling with error from left to right: exact, K (9.9% error), RKHA, and RK Shepp Logan.

**Figure 5:**
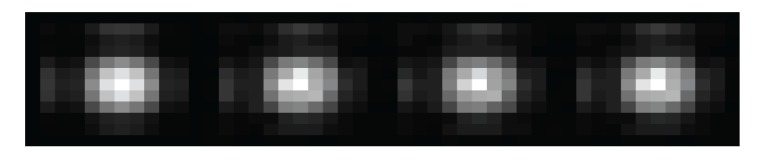
Reconstructed test images for* sinc2d()* with fan sampling with error from left to right: exact, K, RKHA (9.9%), and RK.

The cases for fan and parallel sampling have increasingly higher coherence and increasing numbers of iterations required to meet the 10% error stopping condition. Both test images for fan-beam sampling converge in example Figures 3(c) and 3(d) and show slight benefit from the methods which minimize the coherence, such as RK, RKHA, and RKOS. Example quantitative results for the two cases of fan sampling are shown in (a) Shepp-Logan Table 3 and Figure 4 and (b)* sinc2d()* Table 4 and Figure 5.

Comparison of convergence results to the estimated coherence for the three cases given in Table 5 suggest consistent interpretation. Comparison of percent error and SNR of Tables 1, 2, 3, and 4 indicate the randomization methods have slight advantage under coherent fan sampling, providing increased SNR and lower percent error.

#### 3.1.4. Potential Applications

Since the iterative methods utilize projections, the angles between the optical lines-of-sight (LOS) forming the measurement hyperplanes are of considerable interest in terms of data acquisition and system design. Most methods of computed tomography do not reasonably allow random or orthogonal data sampling of the object of interest.

Therefore, these systems which acquire coherent data may benefit from use of the randomized methods RK, RKHA, or RKOS in data inversions. Typical examples of such systems may include computed tomography in medical and nonmedical X-ray, transmission ultrasound [27, 28], and resonance optical absorption and molecular florescence* in-vivo* imaging [29]. Each of the aforementioned systems are potentially feasible applications of RKHA or RKOS, since in each case, the measurements are path integrated along a mostly nonorthogonal set of electromagnetic LOS's and generally require inversion to obtain the parameters of interest, such mole-fraction or species density.

### 3.2. Experimental Results for Convergence of K, RK, and RKHA

Iterative simulations were performed to estimate the relative convergence rates of methods K, RK, RKHA for the data examples above: random and fan beam sampling. Representative results are shown in Figures 3(a), 3(b), 3(c), and 3(d) for noiseless data measurement scenarios of the standard Shepp-Logan phantom and* sinc2d()* solutions under random and fan-beam sampling simulations. Parallel beam sampling and* rect_sinc2d()* solution vectors were also simulated. The results were consistent with observations reported herein but not included in this report.

#### 3.2.1. Test Images for Algorithms

The Shepp Logan image was generated from the phantom code in reference [22], which is also the source for the fan-beam sampling algorithm. The* sinc2d()* function is defined as

(65) $$\begin{array}{c} sinc2d\left(x,y\right)=\frac{\sin\left(\pi x\right)}{\pi x}\frac{\sin\left(\pi y\right)}{\pi y} \end{array}$$

and was computed as the outer-product of two independent vectors constructed from the included code  sinc.m  in the signal processing package of Octave [21]. The rectangular  rect_sinc2d.m  function was used from source (https://engineering.purdue.edu/VISE/ee438/demos/2D_signals_systems/rect_sinc2d.m) which computes the two-dimensional* sinc()* function from the Fourier transform of the two-dimensional rectangle function.

#### 3.2.2. Image Reconstruction Error

A comparison of the toy phantoms was performed based upon the estimated signal to noise ratio and total percent error versus iteration. Percent error and SNR were estimated for the Shepp-Logan phantom and the artifact based upon first method to obtain stopping error at 10% percent normalized error.

The normalized error is defined as

(66) $$\begin{array}{c} \epsilon_{k}=\frac{{\left\|x-x_{k}\right\|}_{2}}{{\left\|x-x_{0}\right\|}_{2}}, \end{array}$$

and estimate the signal to noise ratio (SNR) is estimated as 1/*ϵ*_*k*_ with the **x**_0_ vector set to the the zero vector.

### 3.3. Experimental Results for Complexity Estimates

**Figure 6:**
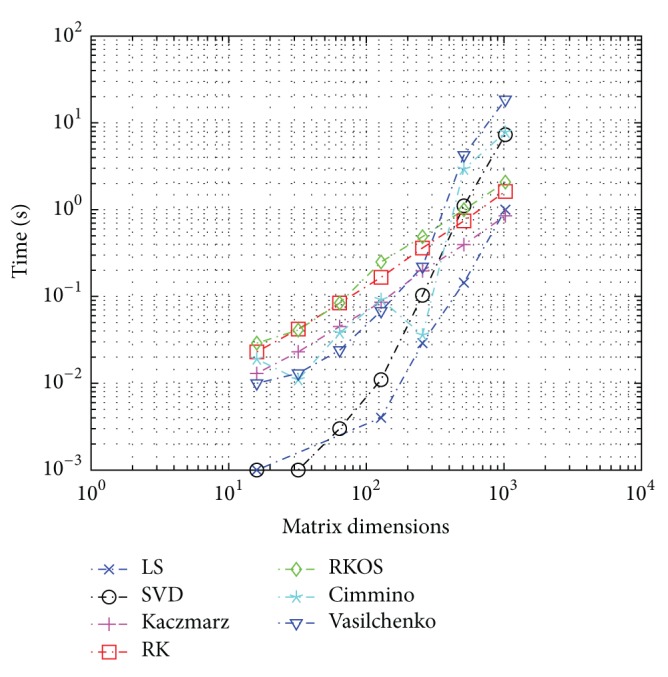
Relative computational time using dedicated custom real-time Linux kernel 3.12.5 patched versus square matrix dimensions *N* = 2^*q*^  ∀*q* ∈ [4,…, 10]; CPU is shielded and frequency locked for single process and single core (one of eight) 800 MHz Intel 64-bit i7.

**Figure 7:**
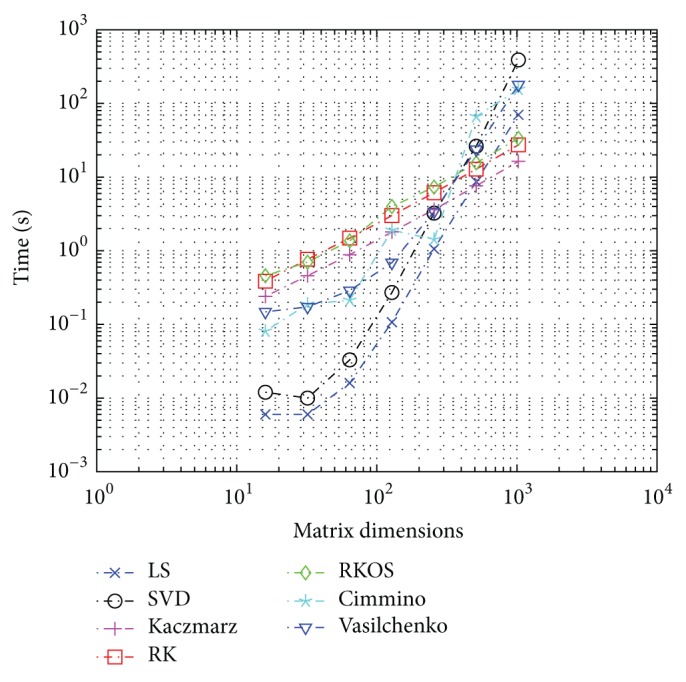
Relative computational time for embedded microprocessor dedicated task custom Linux kernel 3.12.7 ARMv7 relative computational time versus matrix dimensions *N* = 2^*q*^  ∀*q* ∈ [4,…, 10]; CPU ARM frequency locked kernel only process stack 840 MHz 32-bit.

Timings and plots of the methods were computed for a range of matrix sizes and plotted on log-log plots as shown in Figures 6 and 7, with measured run-times for 64-bit and 32-bit, respectively. For each method, the size of *A* matrix was modified and average time to complete was measured. The data matrix was chosen to the be* hadamard()* function for ease of implementation and reference. The solution vector was chosen as a random point in the binomial distribution with *p* = 0.5 using the* binornd()* function.

It is notable that the theoretical complexity of noise limited Kaczmarz is *O*(*N* ^2^) but the slope of the timings is closer to 1.1 than 2.0. This is attributed to relatively small matrix sizes, and it is expected that the complexity asymptotically approaches ~2.0 for large *N*.

The theoretical and numerical estimates for complexity are shown in Tables 6 and 7.

## 4. Conclusions

A new iterative selection rule based upon the relative central angle (RKHA) shows enhanced convergence in measurements which contain coherence. However, the method requires a computational penalty related to the dot-products of all to all rows, which may be overcome by* a priori* determination. A new block method using constructed orthogonal subspace projections provides enhanced tolerance to measurement coherence, but may be affected by noise at least as much as simple Kaczmarz. The exponential convergence is accelerated by the *P*/*N* term and is computationally feasible for small *P* relative to *N*.

The theoretical convergence rates of above subspace methods were demonstrated using statistical IID assumptions or cyclical projections using the formalism of Galantai. Numerical results were presented from simulations of algorithm convergence under measurement distributions for fan and parallel X-ray beams, as well as random uniform sampling.

Relative performance benchmarks for complexity were obtained for typical hardware and software for various methods of well-known Kaczmarz algorithms. As expected, the Kaczmarz methods out-perform other methods, such as least-squares and singular value decomposition. The performance of embedded 32-bit ARM CPU architecture was sufficient to demonstrate functional capability over a range of low-power application environments such as mobile medicine platforms.

The new angle-based method (RKHA) and orthogonal block (RKOS) inversion method demonstrated herein showed quantitative convergence improvement consistent with increasing orthogonality and decreasing coherency of measurements. Future designs for tomography should consider optimization of angular sampling distributions in addition to other factors, such signal to noise ratio, as important system parameters, since these criteria ultimately affect the spatial-temporal resolution and uncertainty for a given number of samples per unit volume.

## Figures and Tables

**Algorithm 1:**
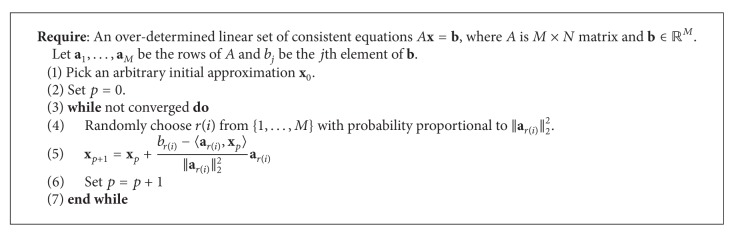
Randomized Kaczmarz (of [4]).

**Algorithm 2:**
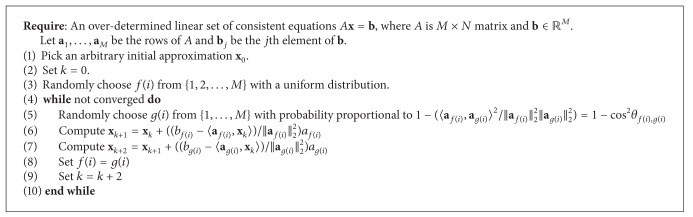
Randomized Kaczmarz Hyperplane Angles.

**Algorithm 3:**
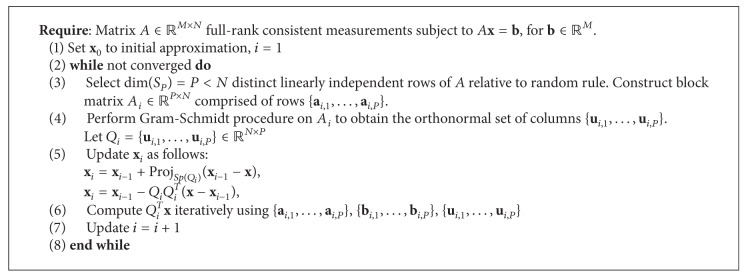
*P*-Subspace Kaczmarz Projections.

**Listing 1:**
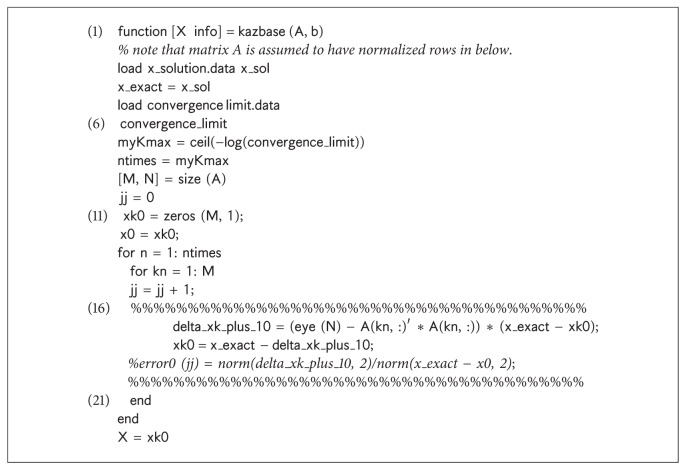
Reference Kaczmarz Baseline.

**Table 1:** Random sampling of Shepp-Logan SNR and percent error at stopping.

Performance estimates	Kaczmarz	RKHA	RK
SNR	10.1	5.60	4.5
% error	9.9	19.9	22.1

**Table 2:** Random sampling of *sinc2d()* SNR and percent error at stopping.

Performance estimates	Kaczmarz	RKHA	RK
SNR	10.0	5.6	5.2
% error	9.9	17.6	19.1

**Table 3:** Fan beam sampling of Shepp Logan, SNR, and percent error at stopping.

Performance estimates	Kaczmarz	RKHA	RK
SNR	10.0	9.95	9.5
% error	9.9	10.0	10.5

**Table 4:** Fan beam sampling of* sinc2d()* SNR and percent error at stopping.

Performance estimates	Kaczmarz	RKHA	RK
SNR	7.1	10.0	9.2
% error	14.0	9.9	10.7

**Table 5:** Typical coherence estimates for *N* = 100, *M* = 200 for random *randn()* and *N* = 100, *M* = 222 for fan *fanbeamtomo()* and parallel *paralleltomo*().

Coherence versus measurement method	Random	Fan	Parallel
Coherence (64)	.4	1.0	1.0
Average value of $G_{i,j}=\langle {\hat{a}}_{i},{\hat{a}}_{j}\rangle \;(1\leq i\neq j\leq M)$	−.0013	.06	.18
Median value of $G_{i,j}=\langle {\hat{a}}_{i},{\hat{a}}_{j}\rangle \;(1\leq i\neq j\leq M)$	−0.0009	0	.12

**Table 6:** Numerical estimates for computational complexity using naive codes.

Computational complexity	LS	SVD	Kaczmarz	RK	RKOS
Theoretical	*O*(*N* ^3^)	*O*(*N* ^3^)	*O*(*N* ^2^)	*O*(*N* ^2^)	*O*(*N* ^2^)
Numerical estimates	2.7	3.1	1.1	1.1	1.1

**Table 7:** Numerical estimates for computational complexity using naive codes.

Computational complexity	Cimmino	Vasilchenko	Ref. *P*1	Ref. *P*2	Ref. Kaczmarz
Theoretical	*O*(*N* ^2^)	*O*(*N* ^2^)	*O*(*N* ^2^)	*O*(*N* ^3^)	*O*(*N* ^2^)
Numerical estimates	1.6	2.1	2	3.2	1.1

## References

[1] Kaczmarz S. (1993). Approximate solution of systems of linear equations. *International Journal of Control*.

[2] Gordon R., Bender R., Herman G. T. (1970). Algebraic reconstruction techniques (art) for three-dimensional electron microscopy and x-ray photography. *Journal of Theoretical Biology*.

[3] Herman G. T. (2009). *Fundamentals of Computerized Tomography: Image Reconstruction from Projections*.

[4] Strohmer T., Vershynin R. (2009). A randomized Kaczmarz algorithm with exponential convergence. *The Journal of Fourier Analysis and Applications*.

[5] Needell D. (2010). Randomized Kaczmarz solver for noisy linear systems. *BIT Numerical Mathematics*.

[6] Needell D., Ward R. (2013). Two-subspace projection method for coherent overdetermined systems. *The Journal of Fourier Analysis and Applications*.

[7] Needell D., Tropp J. A. (2014). Paved with good intentions: analysis of a randomized block Kaczmarz method. *Linear Algebra and its Applications*.

[8] Chen X., Powell A. M. (2012). Almost sure convergence of the Kaczmarz algorithm with random measurements. *Journal of Fourier Analysis and Applications*.

[9] Galántai A. (2005). On the rate of convergence of the alternating projection method in finite dimensional spaces. *Journal of Mathematical Analysis and Applications*.

[10] Galántai A. (2004). *Projectors and Projection Methods*.

[11] Deutsch F., Hundal H. (1997). The rate of convergence for the method of alternating projections, II. *Journal of Mathematical Analysis and Applications*.

[12] Brezinski C., Redivo-Zaglia M. (2013). Convergence acceleration of Kaczmarz's method. *Journal of Engineering Mathematics*.

[13] Smith K. T., Solmon D. C., Wagner S. L. (1977). Practical and mathematical aspects of the problem of reconstructing objects from radiographs. *Bulletin of the American Mathematical Society*.

[14] Bai Z.-Z., Liu X.-G. (2013). On the Meany inequality with applications to convergence analysis of several row-action iteration methods. *Numerische Mathematik*.

[15] Censor Y., Herman G. T., Jiang M. (2009). A note on the behavior of the randomized Kaczmarz algorithm of Strohmer and Vershynin. *Journal of Fourier Analysis and Applications*.

[16] Wallace T., Sekmen A. Acceleration of Kaczmarz using subspace orthogonal projections.

[17] Golub G. H., van Loan C. F. (1996). *Matrix Computations*.

[18] Meyer C. (2000). *Matrix Analysis and Applied Linear Algebra*.

[19] Yanai H., Takeuchi K., Takane Y. (2011). *Projection Matrices, Generalized Inverse Matrices, and Singular Value Decomposition*.

[20] Strohmer T., Vershynin R. (2009). A randomized Kaczmarz algorithm with exponential convergence. *Journal of Fourier Analysis and Applications*.

[21] Eaton J., Rik W., Bateman D., Hauberg S. (2014). *GNU Octave Version 3.8.1 Manual: A High-Level Interactive Language for Numerical Computations*.

[22] Hansen P. C., Saxild-Hansen M. (2012). AIR-tools—a MATLAB package of algebraic iterative reconstruction methods. *Journal of Computational and Applied Mathematics*.

[23] Vasil'chenko G. P., Svetlakov A. A. (1980). A projection algorithm for solving systems of linear algebraic equations of high dimensionality. *USSR Computational Mathematics and Mathematical Physics*.

[24] Marsaglia G., Tsang W. W. (2000). The ziggurat method for generating random variables. *Journal of Statistical Software*.

[25] Donoho D. L., Elad M., Temlyakov V. N. (2006). Stable recovery of sparse overcomplete representations in the presence of noise. *IEEE Transactions on Information Theory*.

[26] Welch L. R. (1974). Lower bounds on the maximum cross correlation of signals (corresp.). *IEEE Transactions on Information Theory*.

[27] Birk M., Dapp R., Ruiter N. V., Becker J. (2014). GPU-based iterative transmission reconstruction in 3D ultrasound computer tomography. *Journal of Parallel and Distributed Computing*.

[28] Peterlík I., Ruiter N., Stotzka R. Algebraic reconstruction technique for ultrasound transmission tomography.

[29] Ntziachristos V. (2006). Fluorescence molecular imaging. *Annual Review of Biomedical Engineering*.

